# Ensemble Feature Learning of Genomic Data Using Support Vector Machine

**DOI:** 10.1371/journal.pone.0157330

**Published:** 2016-06-15

**Authors:** Ali Anaissi, Madhu Goyal, Daniel R. Catchpoole, Ali Braytee, Paul J. Kennedy

**Affiliations:** 1 Centre for Quantum Computation & Intelligent Systems (QCIS), Faculty of Engineering and Information Technology (FEIT), University of Technology Sydney (UTS), Broadway NSW 2007, Australia; 2 The Tumour Bank, Children’s Cancer Research Unit, The Children’s Hospital at Westmead, Locked Bag 4001, Westmead NSW 2145, Australia; Harbin Institute of Technology Shenzhen Graduate School, CHINA

## Abstract

The identification of a subset of genes having the ability to capture the necessary information to distinguish classes of patients is crucial in bioinformatics applications. Ensemble and bagging methods have been shown to work effectively in the process of gene selection and classification. Testament to that is random forest which combines random decision trees with bagging to improve overall feature selection and classification accuracy. Surprisingly, the adoption of these methods in support vector machines has only recently received attention but mostly on classification not gene selection. This paper introduces an ensemble SVM-Recursive Feature Elimination (ESVM-RFE) for gene selection that follows the concepts of ensemble and bagging used in random forest but adopts the backward elimination strategy which is the rationale of RFE algorithm. The rationale behind this is, building ensemble SVM models using randomly drawn bootstrap samples from the training set, will produce different feature rankings which will be subsequently aggregated as one feature ranking. As a result, the decision for elimination of features is based upon the ranking of multiple SVM models instead of choosing one particular model. Moreover, this approach will address the problem of imbalanced datasets by constructing a nearly balanced bootstrap sample. Our experiments show that ESVM-RFE for gene selection substantially increased the classification performance on five microarray datasets compared to state-of-the-art methods. Experiments on the childhood leukaemia dataset show that an average 9% better accuracy is achieved by ESVM-RFE over SVM-RFE, and 5% over random forest based approach. The selected genes by the ESVM-RFE algorithm were further explored with Singular Value Decomposition (SVD) which reveals significant clusters with the selected data.

## Introduction

Designing a classification model for patients, based on their gene expression profile, to capture their variation based upon their subtype of disease is still a focus of many researchers working in the field of bioinformatics [1] [2] [3]. The key difficulties of building this model are the vast number of gene expression measurements being generated for each patient through high throughput genomic technologies. These data are typically very complex, noisy and highly dimensional. Moreover, many cohorts have the imbalanced classes problem which raises challenges in dealing effectively with this kind of data.

Consequently, feature selection has become a prerequisite in many genomic applications [4]. It selects a small subset of useful attributes that capture the necessary information to explain the differences between patients based upon their subtype of disease. By selecting these attributes, the dimensionality of the data is decreased and the ability to visualise and understand the data can be better realised whilst maintaining an informatively ‘rich’ set of features to provide higher quality results. Other benefits of performing attribute selection include the capability to build robust and compact models using only a subset of the original attributes.

Saeys et al [5] define a taxonomy of feature selection techniques which divides feature selection into three approaches: filter, wrapper and embedded. Filter based algorithms are independent of the classifiers. That is, genes are selected based on an evaluation criterion such as looking at the intrinsic properties of the data regardless of the classifier [6]. The wrapper and embedded approaches, on the other hand, select a subset of genes based on the classification performance of a specific model. Wrapper methods follow a search strategy to find optimal feature subset tailored to a particular classification model, while embedded methods use the evaluation of classification model to perform feature selection. Embedded methods are less computationally expensive than wrapper methods and less prone to over-fitting, especially in high-dimensional spaces [5].

In the present study, we use the embedded methods with Support Vector Machine (SVM) [7] classifiers as the base learning model. Support Vector Machine (SVM) is a classifier using a decision boundary to separate two classes defined by solving a quadratic optimization problem. SVM finds an optimal solution that maximizes the distance between the hyperplane and the most critical training samples. The decision boundary is then specified by a subset of critical training samples named support vectors that lie on the edge. SVM extends to multi-class classification using several methods [8] [9] [10]. SVM has been extensively and effectively used in many bioinformatic applications because its design is well suited to genomic data. SVM has the ability to work in high dimensional space compared to the small number of samples [11]. Several embedded methods have been proposed for feature selection using SVM classifier acting as a base learning algorithm to decide which feature has to be eliminated [12] [8] [13]. A dominant approach among them is the SVM-RFE selection method proposed by Guyon et al [13].

Besides embedded methods, ensemble methods [14] are also effective learning techniques that have been introduced to improve overall prediction and feature selection accuracy. In this approach, several classification models are combined to make the decision for elimination of features instead of choosing one particular classifier. A good example is random forest [15] which combines random decision trees with bagging to improve overall feature selection and classification accuracy. Moreover, ensemble techniques have the advantage of handling the problems of small sample size and high-dimensionality associated with gene expression datasets. They also reduce the potential of over fitting the training data. With ensemble methods, the training dataset may be used more efficiently and a feature selection process can be achieved using multiple prediction models each with a different sample subset.

Surprisingly, the adoption of ensemble methods in support vector machine has received attention recently but mostly on classification not gene selection [2] [16] [17]. For instance, Zou et al [17] employ a machine learning approach based on a novel ensemble classifier to predict cytokines. Similarly, Ding at al [18] use ensemble SVM classifier to achieve better predictions, but without doing any feature selection. Abeel et al [2] proposed ensemble feature selection methods based on SVM-RFE. They merely employ ensemble methods as multiple executions of SVM-RFE algorithm in order to increase the stability of the selected features. However, our approach employs ensemble methods through constructing multiple SVM models at each iteration of SVM-RFE. The rationale behind this idea is to take the strengths of random forest, such as ensemble and bagging, and apply it onto SVM. Valentini et al [19] thoroughly analyse the bias and variance of SVM for the development of SVM-based ensemble methods. The authors show that the bias-variance decomposition offers a hypothesis to develop ensemble methods using SVMs as base learners [19].

In the following, we introduce an ensemble SVM (ESVM) for gene selection that follows the ensemble and bagging concepts of random forest and adopts the backward elimination strategy which is the rationale of recursive feature elimination algorithm [13]. The idea behind this is that, building an ensemble of SVM models in each iteration of the SVM-RFE using a randomly drawn subset of the training set, will produce different feature rankings which will be aggregated as one ensemble vote. As a result, the decision for elimination of features is based upon the votes of multiple SVM models instead of choosing one particular model. Moreover, this approach will allow us to handle the problem of imbalanced datasets by constructing roughly balanced bootstrap samples or bootstrap samples biased to the minority class.

## Materials and Methods

### Ensemble SVM

This paper introduces a new algorithm for gene selection from microarray gene expression datasets called Ensemble SVM-RFE (ESVM-RFE). ESVM-RFE is an embedded feature selector algorithm that follows a backward feature elimination method. Our base learning classifier is a linear SVM which has been thoroughly investigated and benchmarked against a variety of state-of-the-art methods [15] [20]. SVM is involved in the process of determining what attributes to remove at each step since SVM generates a weight for each feature according to the absolute value of their weight in the hyperplane. These weights can be used to rank the features from most important to least important, which is the principle for SVM-RFE algorithm [13].

Consequently, we propose an ensemble feature learning method which relies on the bagging approach with SVM classifiers as the base learning algorithm. Following this approach, several feature rankings are combined to make a principled decision for elimination of features at each iteration in the SVM-RFE instead of choosing one particular feature ranking of SVM for a particular sample.

### Algorithm of ESVM-RFE

The algorithm starts with the entire set of features in the dataset, and at every iteration, we train an ensemble SVM by taking bootstrap samples from the original training dataset. In order to mitigate the effect of class imbalanced data on classification, we take approximately equal numbers of samples from each class

The estimated feature weights from each SVM are aggregated to form one final evaluation of the ensemble. Subsequently, we sort the features according to their estimated weights in decreasing order. The least important features with the smallest weight are eliminated and another ensemble is re-constructed but restricted to the remaining set of features. This process is repeated iteratively until a desired number of features is left, where this number is a user defined variable (see Algorithm ESVM-RFE).

**Algorithm ESVM-RFE** (data,class,b,E,d,bagSize)

**Require**: *surviveIndexes* = *seq*(1 : *ncol*(*data*))

**Require**: *n* = *nrow*(*data*)

 **while** *length*(*surviveIndexes*) ≥ *d* **do**

  *m* = *length*(*surviveIndexes*)

  *survive* = *m* − *m* × *E* {survive: number of features to select in the current iteration}

  *ensRes* = *matrix*(*n*, *b*) {ensRes: feature’s weight of each SVM model}

  **for** *i* = 1 : *b* **do**

   *bag* ← *bootstrap*(*data*,*bagSize*)

   *bagClass* ← *bootstrap*(*class*,*bagSize*)

   *model* ← *svm*(*bag*[,*survivingIndexes*],*bagClass*)

   *weightVector* ← *transpose*(*model*$*coefs*)%*%*model*$*SV* {Compute the weight vector}

   *featureWeight* ← weightVector*weightVector {Compute ranking criteria}

   *ensRes* ← *merge*(*ensRes*,*featureWeight*) {Accumulate feature’s weight}

  **end for**

  *totalWeight* = *rowSum*(*ensRes*) {Aggregate feature’s weight}

  *sortedWeight* ← *sort*(*totalWeight*) {Sort the total feature’s weight by decreasing order}

  *sortedIndexes* ← *index*(*sortedWeight*)

  *surviveIndexes* ← *surviveIndexes*[*sortedIndexes*[1 : *survive*]] {Eliminate features with smallest weight}

 **end while**

 *selectedData* = *data*[,*surviveIndexes*]

The R package e1071 that contains the algorithm SVM was used in this paper to implement our ESVM-RFE algorithm which takes six parameters. Two parameters highly affect the computational complexity of our ensemble SVM: *E*, the proportion of features to eliminate at each iteration and *b*, the number of SVMs to train in each ensemble. Decreasing *E* increases the computational cost since less features are removed at each step but perhaps provides more well-mannered elimination. In normal SVM-RFE [13], one feature is eliminated at each step, but the authors reported that the algorithm can be generalised to remove more than one feature per step to improve computational time. Similarly, our ESVM-RFE can be also generalized to remove more than one feature per step. Accordingly, because we are constructing ensemble SVM in each step and in order to speed up our process, we chose *E* = 10% by default [21].

With respect to the parameter *b*, it is associated with our main aim to obtain a diverse set of feature rankings by drawing different bootstrap samples of the training data to train *b* SVMs. In general, the more models you train the better results you get. However, at a certain point the benefit in prediction performance from learning more SVMs will be lower than the cost in computation time for learning these additional SVMs. Furthermore, learning a large number of SVMs may produce redundant feature rankings because SVM-RFE procedure is deterministic. In this paper, we chose to generate 40 bags by default from the training data. The default values of these two parameters (*b* and *E*) were based on earlier work [21], where it was demonstrated that these parameter settings provided a good default.

Another parameter is the *bagSize* vector which contains the number of samples to take from each class of the training dataset in order to train each SVM in the ensemble. This parameter plays an important role in handling the problem of class imbalanced data. ESVM-RFE algorithm has the capacity to mitigate the effect of imbalanced classes on gene selection through constructing bootstrap samples to build the ensemble SVM. So that, at the same time as we are extracting random bootstrap samples from the original training dataset, we induce our sampling approach to generate a roughly balanced bootstrap samples.

These defined parameters are passed to ESVM-RFE algorithm along with the parameters *data*, a data matrix contains the dataset, *class*, the response vector with one label for each row *data* and *d*, the desired number of features.

### Datasets

We have applied our feature selection approach on five microarray datasets using ESVM. The main characteristics of these datasets are summarised in Table 1. They share common characteristics such as a very small number of samples compared to thousands of genes and some of them are imbalanced. The Affymetrix childhood leukaemia dataset is generated from the U133A platform and it is collected from The Children’s Hospital at Westmead. For more information, see S1 Text.

**Table 1:** Microarray gene expression datasets.

Datasets	Number of classes	Number of features	Number of training samples	Number of testing samples	Profiles
Childhood Leukaemia	2	22,277	45	15	Relapse/Non Relapse
NCI	8	5,244	46	15	8 phenotypes
Colon cancer	2	2,000	46	16	Cancer/Normal
Breast2 cancer	2	4,869	58	19	2 phenotypes
Breast3 cancer	3	4,869	72	23	3 phenotypes

This childhood leukaemia gene expression dataset contains data for 60 patients with expression values for 22,277 probes. This dataset has associated clinical data which contains laboratory test results for each patient in addition to the general information about patients such as treatment received, sex, age, date of birth, etc. Patient information was anonymized and de-identified prior to analysis. The clinical data also contains outcomes for each patient such as relapse status. This clinical information about relapse status is used in this study as a class label for each patient in order to perform attribute selection. A stratified random sampling is applied on the gene expression dataset in order to take quarter of the data as a test dataset having the same distribution of the patients to the training data. For that we have used the R package sampling which has the method balancedstratification. The training and test datasets are composed of 45 and 15 patients, respectively. The distribution of patients in each dataset is shown in Table 2.

**Table 2:** The number of patients in the training and test datasets for childhood leukaemia dataset.

	Relapse	Non-Relapse	Total
Training dataset	15	30	45
Test dataset	6	9	15

The second Affymetrix microarray dataset used in this study is the Colon cancer dataset [22] which contains 2000 expression values for 62 samples. Each sample indicates whether or not it came from a tumour biopsy [22]. This dataset allows us to objectively compare our result with earlier published results as it has been used in many different research papers [23] [24] [25].

The third dataset, the National Cancer Institute cancer cell line dataset, is also a well-studied publicly available microarray benchmark collected by Ross et al [26] and is produced using Affymetrix HG-U133A chips. The dataset consists of 61 samples that are classified into eight categories. Each sample is measured over 5,244 gene expression values. This dataset is complex as it is composed of multiple classes with a low number of samples in some classes which raise many challenges for the analysis.

The fourth dataset used in this study is related to breast cancer and was downloaded from [http://www.rii.com/publications/2002/vantveer.htm] [27] as cited in [28]. The dataset measures 4,869 gene expression values corresponding to 33 patients that developed distant metastases within 5 years, 44 that remained disease-free for over 5 years, and 18 with BRCA1 germline mutations and 2 with BRCA2 mutations). Similarly to the authors in [28], we excluded the 2 patients with the BRCA2 mutation because of the small sample size. The breast cancer dataset was used both for two class comparison (those that developed metastases within 5 years vs. those that remain metastases free after 5 years) and for three group comparisons by adding the patients with BRCA2 germline mutations. The two class dataset is named Breast2 containing 78 patients, and the three classes dataset is named Breast 3 with a total of 95 patients. The five reported datasets are z-score normalized.

## Results and Discussion

We report here the experimental evaluations on the five cancer microarray datasets. One of the most important experiments is to compare our newly proposed ensemble SVM method to the state-of-the-art SVM-RFE by analysing the classification performance for each of the five cancer datasets. All the experiments were run separately on an unloaded machine (with 3.4GHz i7 processors and 8GB memory).

### Experiments on Childhood Leukaemia Dataset

We have initially applied our algorithm on the childhood leukaemia dataset. We run the first experiment by taking 40 bootstrap samples from the training dataset. The ranking criteria for each feature from the 40 sub-samples are added linearly and the features with the smallest weight are discarded at each iteration. The result of this experiment is a set of features ranked in decreasing order. To select the best top features, we used Leave One Out cross validation. A 100% AUC accuracy is achieved on the training dataset with number of features equal to 36.

**Fig 1:**
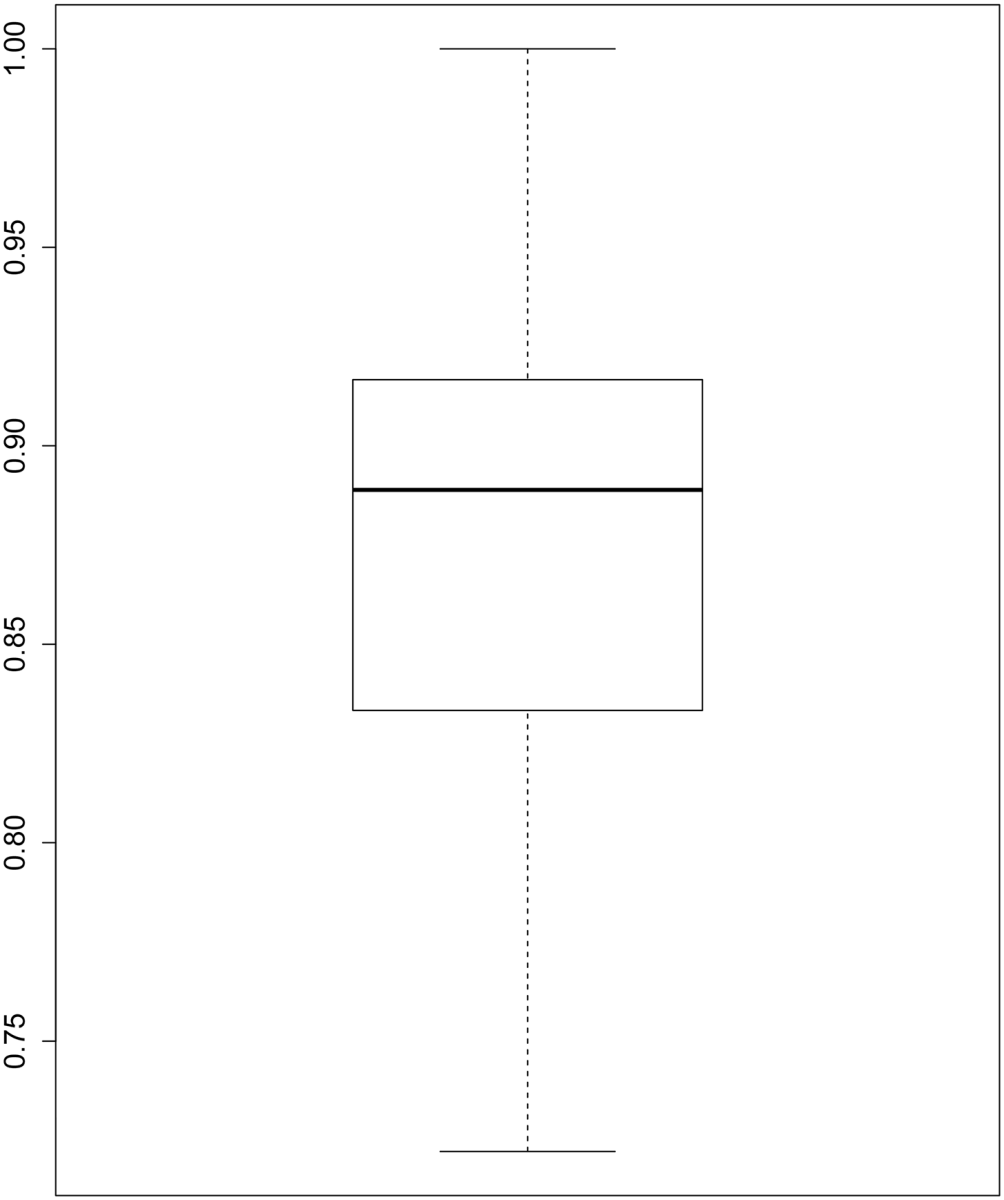
Box plot of AUC accuracies. Box plot of AUC accuracies of the Childhood Leukaemia test dataset evaluated by SVMs trained on 100 bootstrap samples.

The next experiment is to estimate the AUC accuracy of the test dataset using the selected data of 36 attributes by ESVM-RFE. For that, we have used the.632+ bootstrap method [29] [30] with 100 bootstrap samples. For each bootstrap sample, we have estimated the AUC of the test dataset evaluated on each SVM model. The results of this experiment is shown in Fig 1. It can be clearly seen that for some bootstrap samples, the AUC accuracy of the test dataset reaches one for the 36 selected data. The Average AUC accuracy of the 100 bootstrap samples is equal to 0.88.

The next experiment compares our achieved results to the result obtained by SVM-RFE. For that, we have applied SVM-RFE on the childhood Leukaemia dataset using the same training and test samples in order to objectively compare the classification performance of the SVM using the features obtained by ESVM-RFE and the features produced by SVM-RFE.

**Fig 2:**
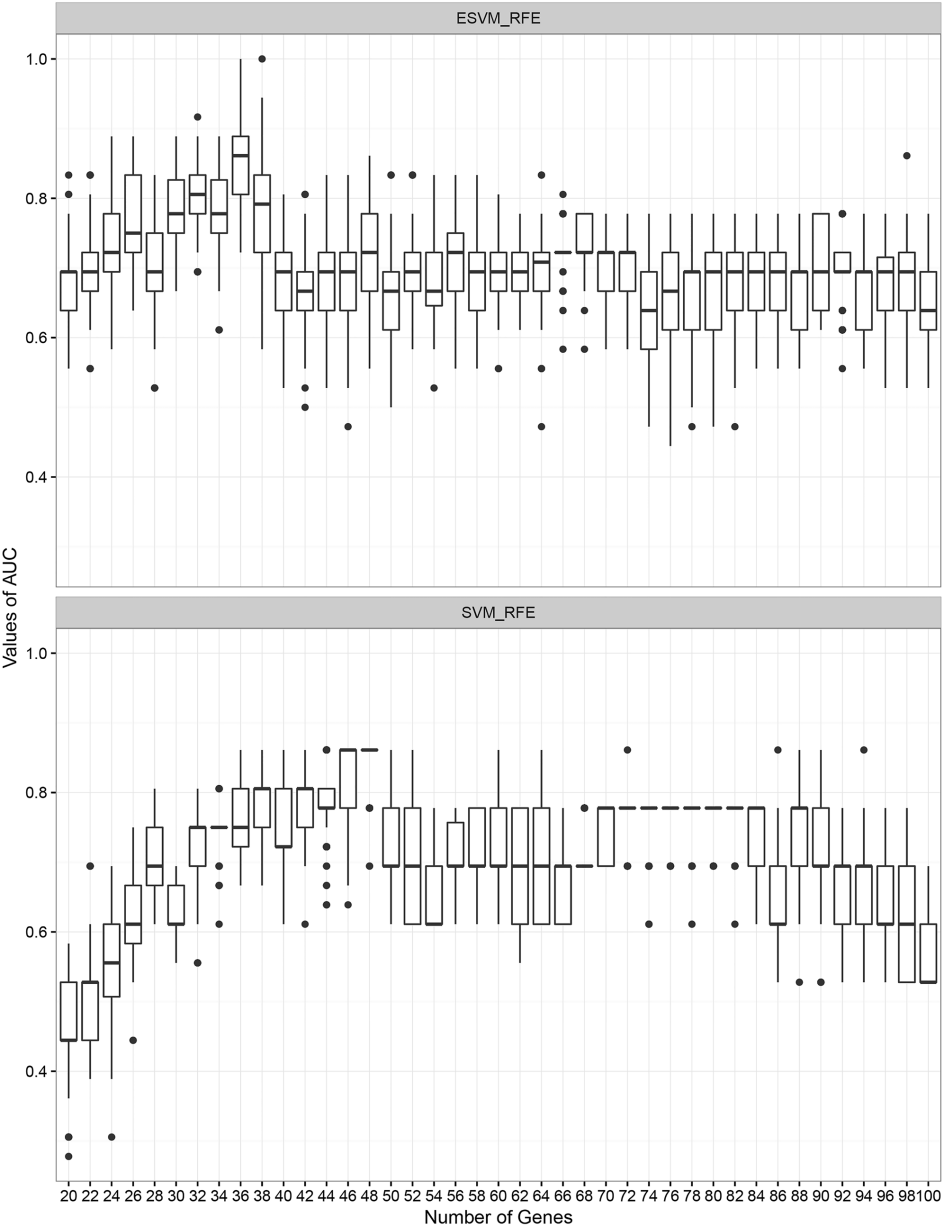
ESVM-RFE vs SVM-RFE. Classification performance comparison between ESVM-RFE and SVM-RFE evaluated using the 0.632+ bootstrap method with 100 bootstrap samples across different number of features.

Initially, we generate 100 bootstrap samples from the training dataset using the 0.632+ bootstrap method to train two SVMs with the selected genes by ESVM-RFE and SVM-RFE. The AUC of the test dataset evaluated on each SVM model is calculated and it suggests that our ESVM-RFE algorithm outperforms SVM-RFE. Fig 2 shows the AUC evaluated on the test dataset across different number of features for ensemble SVM and SVM-RFE. As can be seen, the highest AUC accuracies across the different bootstrap samples are achieved at number of features equal to 36 and 46 for the selected data by ESVM-RFE and SVM-RFE, respectively.

**Fig 3:**
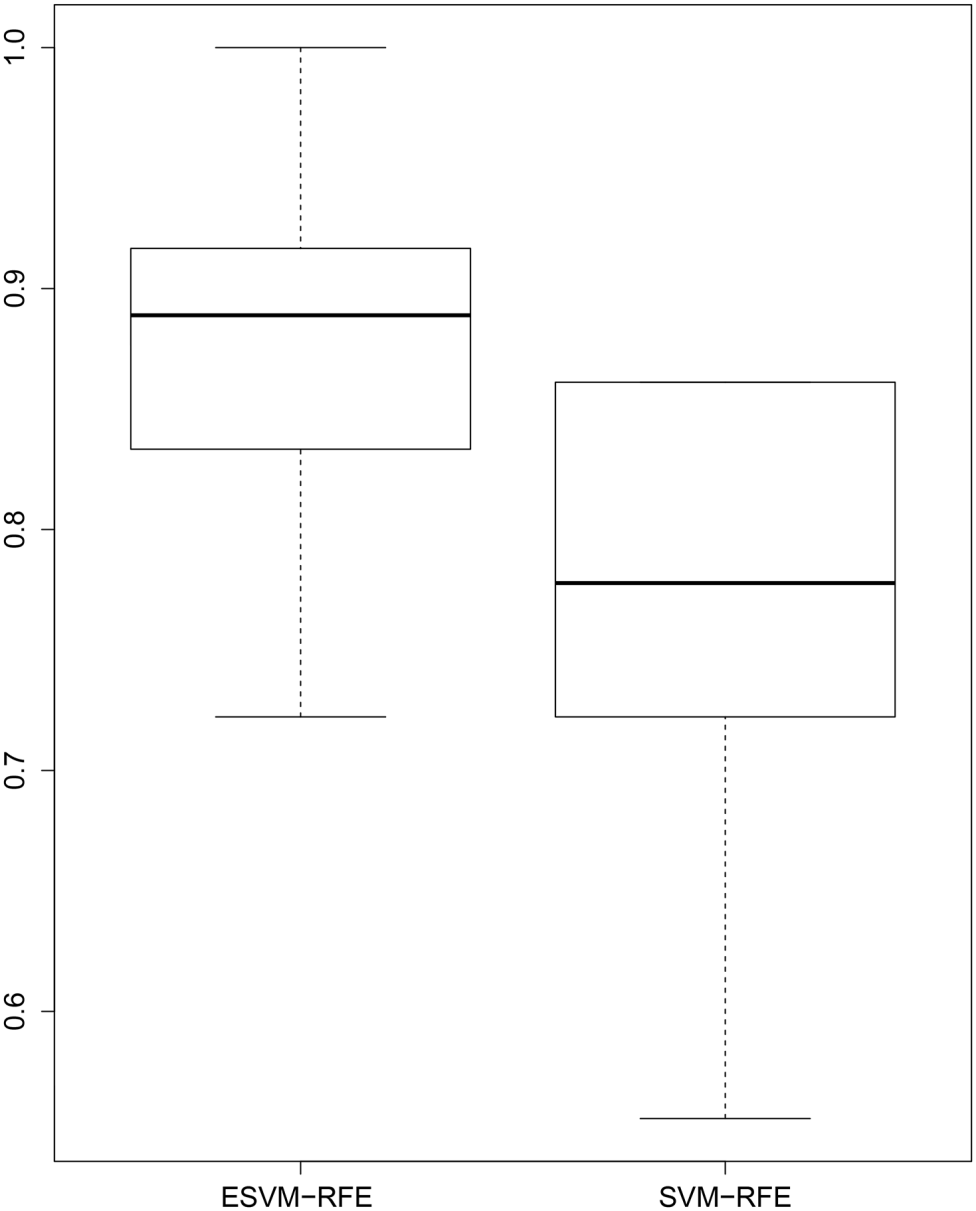
ESVM-RFE vs SVM-RFE. Classification performance comparison between ESVM-RFE and SVM-RFE estimated using the 0.632+ bootstrap method with 100 bootstrap samples at number of features equal to 36 and 46, respectively.

We have further investigated the classification performance of the selected data by ESVM-RFE and SVM-RFE at number of features equal to 36 and 46, respectively. Fig 3 shows the box plot of AUCs of the test dataset by SVM trained on 100 bootstrap samples. The AUC of the selected data by ESVM-RFE significantly outperforms the AUC of selected data by SVM-RFE. The highest AUC accuracy achieved by SVM-RFE is 0.86 in contrast to ESVM-RFE which achieves 100% AUC accuracy for six bootstrap samples and an AUC accuracy greater than or equal to 0.91 for 42 samples. The overall average AUC accuracies of ESVM-RFE and SVM-RFE are 0.88 and 0.79, respectively. Table 3 shows the summary classification performance comparison between ESVM-RFE and SVM-RFE. The paired t-test is also conducted and generates a P-value of 0.03 which indicates a statistically significant different between ESVM-RFE over SVM-RFE.

**Table 3:** The quartile and mean values of AUC accuracies of ESVM-RFE, SVM-RFE and BIRF using Childhood Leukaemia dataset.

	Min	1st Qu	Median	Mean	3rd Qu	Max
ESVM-RFE	0.76	0.85	0.89	0.88	0.94	1
SVM-RFE	0.66	0.77	0.8	0.79	0.86	0.86
BIRF	0.72	0.8	0.85	0.83	0.88	0.92

We have also compared the performance of ESVM-RFE to Random Forest based approach such as Balanced Iterative Random Forest (BIRF) [31]. We have applied BIRF on the childhood Leukaemia dataset using the same training and test samples. Similarly to the authors in [31], we randomly split the dataset, by the number of genes, into multiple datasets having the same count of samples but different number of genes. This process is applied only in the first iteration of the algorithm due to the limitations of BIRF in terms of running time. The average AUC accuracy of the random forest model built on the 67 BIRF-selected features is 0.83. Table 3 shows the summary classification performance of BIRF.

The computational time of these experiments are presented in Table 4. As can be seen, the running time of ESVM-RFE is quite similar to SVM-RFE with *E* = 1% and *B* = 40. But it much faster when *E* is set to 10%. BIRF algorithm, on the other hand, consumes more time compared to ESVM-RFE and SVM-RFE.

**Table 4:** Comparison of ESVM-RFE, SVM-RFE and BIRF in terms of time.

Data	ESVM-RFE (*E* = 10%)	ESVM-RFE (*E* = 1%)	SVM-RFE	BIRF
Childhood Leukaemia	25.851 mins	10.259 hours	9.496 hours	12.208 hours
Colon	1.324 mins	5.328 mins	4.587 mins	8.483 mins
Breast	3.021 mins	2.105 hours	1.799 hours	2.539 hours
NCI	4.362 mins	2.635 hours	2.482 hours	3.029 hours

#### SVD on Childhood Leukaemia Dataset

**Fig 4:**
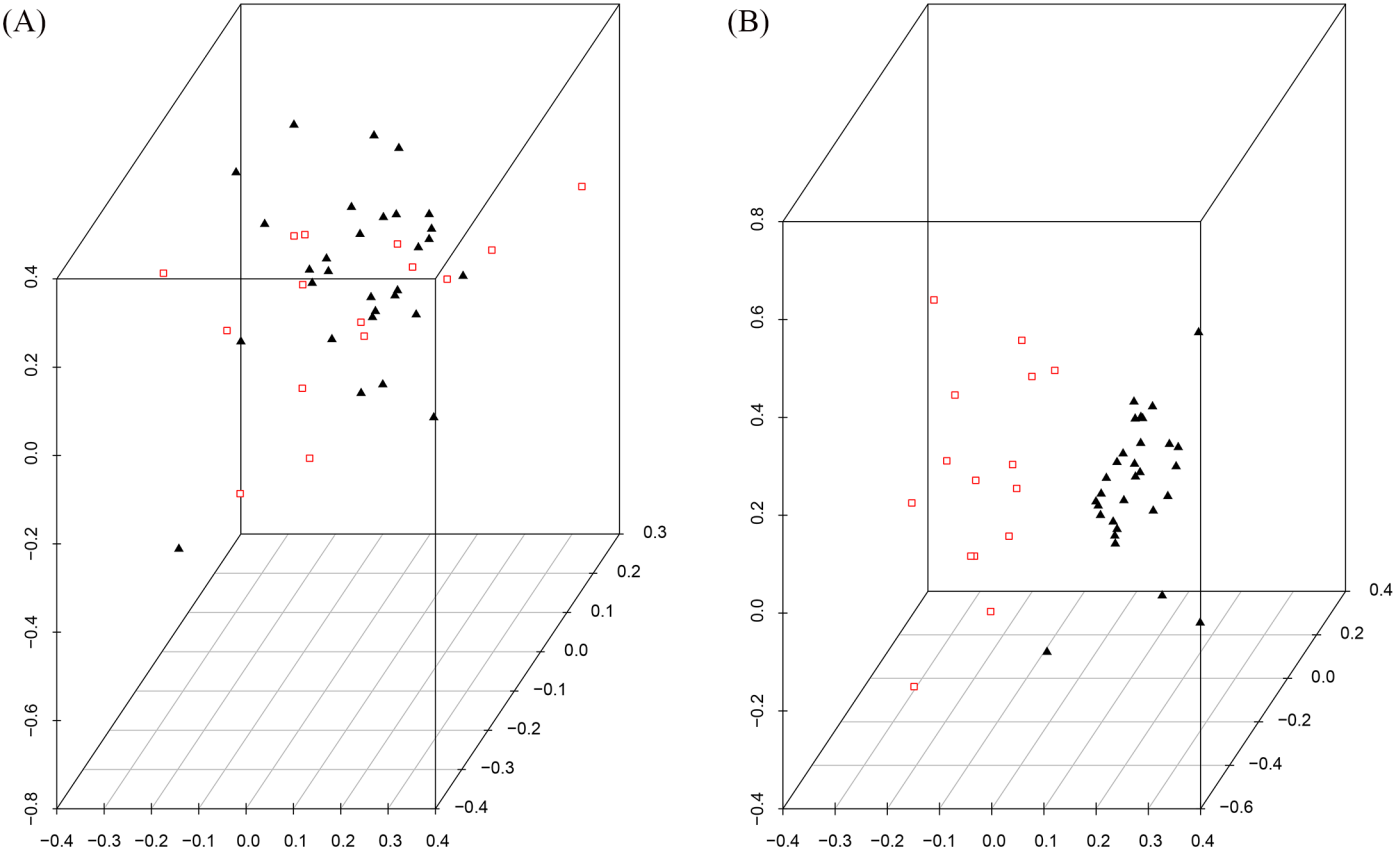
SVD images of childhood Leukaemia data. (a) SVD image of the original training data. (b) SVD image of the selected 36 attributes from the training data, red = Relapse, black = Non Relapse.

Here we look further at the selected attributes from the original childhood leukaemia dataset. For that, we used SVD to see if there is a separation of the selected data based on the class label relapse. We are trying to see if the defined clusters (Relapse/Non Relapse) of the data can be seen now with the selected attributes where it could not be seen in the original data. The resulting SVD of the 36 selected probes is shown in Fig 4. It is clear that there is clustering in this data based on the Relapse label, as we expected from the SVM classification.

**Fig 5:**
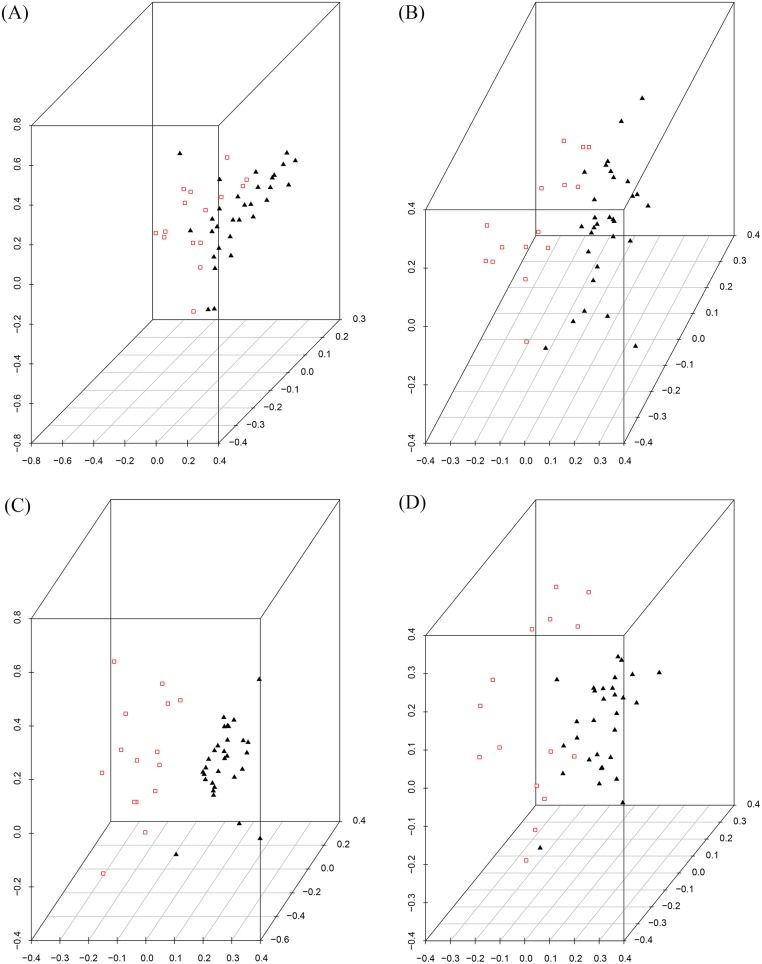
SVD images of childhood Leukaemia data across different number of attributes of the selected data. (a) SVD on 10 attributes, (b) SVD on 25 attributes, (c) SVD on 36 attributes, and (d) SVD on 100 attributes, red = Relapse, black = Non Relapse.

We have applied SVD on the top 10 of the obtained ranked features. The resulting SVD is shown in Fig 5 which demonstrates that there is a grouping in the top 10 however without a clear partition. The top 25 attributes are similar but with just somewhat more separation between the two classes. On the other hand, for the main 36 attributes in Fig 5 we can now see a clear partition between the two classes. The separation between the two clusters has been raised in this subset, along these lines we accept that a large portion of the essential characteristics for relapse are contained in this subset of the data.

This is precisely what we anticipated to discover based upon the SVM results. The separation between the two groups begin to reduce again by applying SVD on the top 100 attributes in Fig 5).

**Fig 6:**
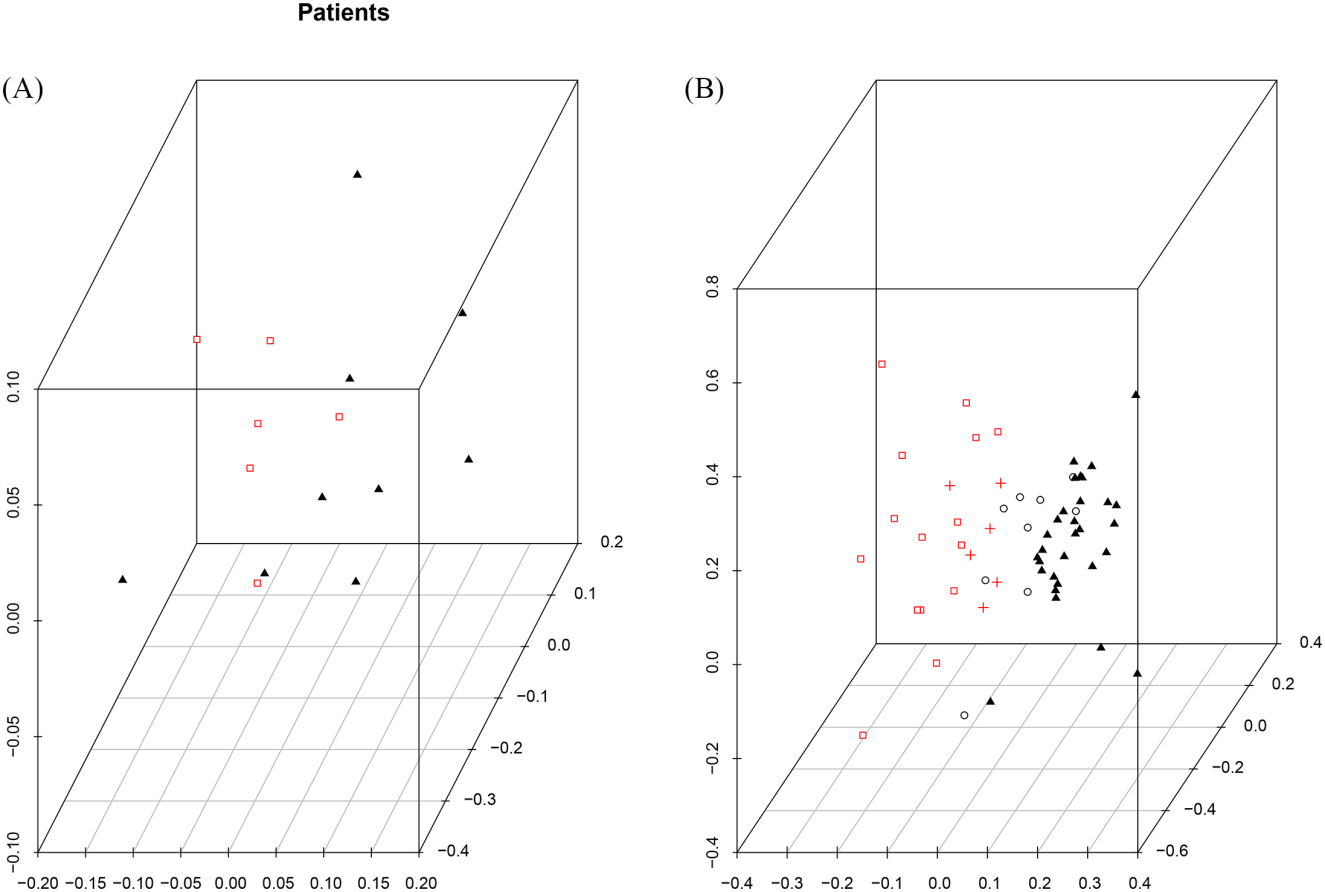
SVD images of childhood Leukaemia test data. (a) SVD image of the test data with the 36 selected attributes. (b) Projection of the test dataset on the low dimensional vector of the selected training data, red = Relapse, black = Non Relapse.

In order to fully understand the classification power of this subset of 36 probes in separating data based on a relapse label, it is necessary to apply this to a new set of patients and see if the desired result is still obtained. Consequently, we applied SVD on the test dataset with the selected 36 attributes. The resulting SVD is shown in Fig 6 which shows that there is a clustering but without a perfect separation. The next step is to project this test data on the selected data from the training dataset. As can be seen from the Fig 6, The new patients are almost grouped with their target clusters which suggest that these selected attributes contain informative probes that can capture the necessary information.

### Experiments on Four Public Microarray Datasets

#### Experiments on Colon Cancer Dataset

Our ensemble SVM algorithm is further validated on the colon cancer dataset. The same iterative procedure is applied here for backward feature elimination and the result of this experiment produces a set of features ranked in decreasing order. The Leave One Out test using the ranked features of the ESVM-RFE gives 100% of AUC accuracy with number of features equal to 11. The evaluation of the test dataset by SVM trained on 100 bootstrap samples using the 0.632+ bootstrap method gives an average value of AUC equal to 0.94.

**Fig 7:**
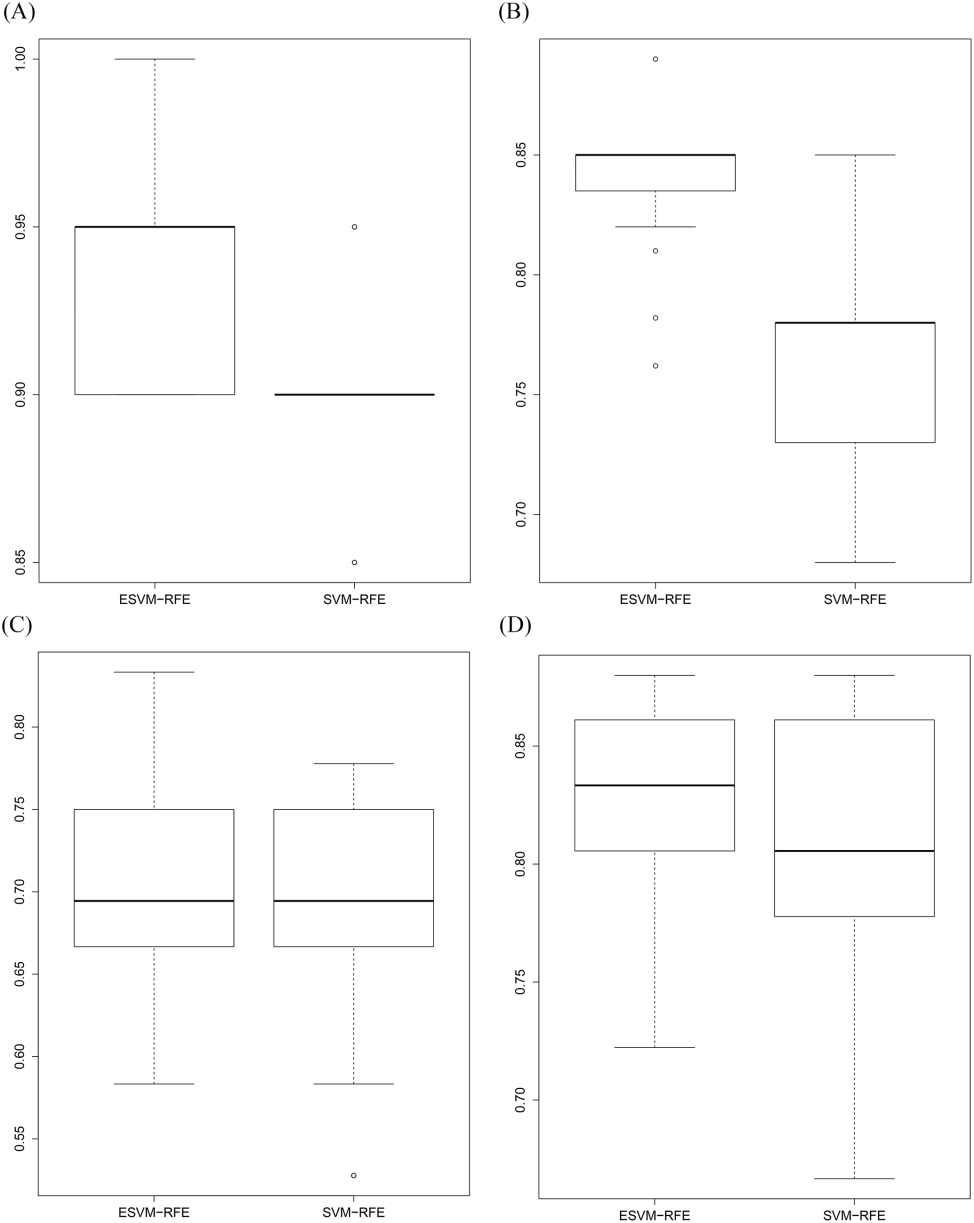
Box plot of AUC accuracies on the four microarray datasets. Comparison of AUC accuracies on the four microarray datasets estimated using the 0.632+ bootstrap method with 100 bootstrap samples. (a) Colon dataset, (b) Breast2 dataset, (c) Breast3 dataset, and (d) NCI dataset.

We have also applied SVM-RFE on the colon dataset with the same samples in the training and test datasets. The Leave One Out test suggests 16 features to be selected from the top ranked features. The average classification performance of SVM-RFE evaluated on the same 100 bootstrap samples gives an average value of AUC equal to 0.89. The AUC values of ESVM-RFE and SVM-RFE across the 100 bootstrap samples are shown in Fig 7. Table 5 shows the summary classification performance comparison between ESVM-RFE and SVM-RFE.

**Table 5:** The quartile and mean values of AUC accuracies of ESVM-RFE, SVM-RFE and BIRF on the four microarray datasets.

Dataset	Method	Min	1st Qu	Median	Mean	3rd Qu	Max
Colon	ESVM-RFE	0.9	0.9	0.95	**0.94**	0.95	1
	SVM-RFE	0.85	0.9	0.9	0.89	0.9	0.95
	BIRF	0.83	0.87	0.92	0.9	0.91	0.98
Breast 2	ESVM-RFE	0.76	0.84	0.85	**0.84**	0.85	0.89
	SVM-RFE	0.67	0.73	0.78	0.77	0.78	0.84
	BIRF	0.68	0.75	0.8	0.79	0.83	0.88
Breast 3	ESVM-RFE	0.56	0.62	0.67	0.66	0.69	**0.78**
	SVM-RFE	0.48	0.63	0.66	0.66	0.68	0.74
	BIRF	0.61	0.63	0.67	**0.68**	0.72	0.76
NCI	ESVM-RFE	0.71	0.78	0.81	**0.81**	0.84	0.89
	SVM-RFE	0.67	0.75	0.79	0.78	0.82	0.89
	BIRF	0.68	0.73	0.77	0.76	0.81	0.83

We have also compared our results to the results obtained by [2] using ensemble feature selection methods (SVM-RFE). The maximum AUC accuracy achieved by [2] is equal to 0.87 where ESVM-RFE achieves an average AUC accuracy equal to 0.94.

As our method is inspired by the success of ensemble approach of feature selection with random forest. We report here the AUC accuracies results obtained by two benchmarks feature selection techniques based random forest, feature importance measures of Random Forests [28] (0.87) and BIRF [31] (0.9) (see Table 5).

#### Experiments on Breast Cancer Dataset

The two breast cancer datasets (two and three classes) are involved in these experiments. We performed initial experiment on breast 2 cancer dataset. This experiment produced a set of features sorted in decreasing order. The Leave One Out test using the ranked features gives 0.97 of AUC accuracy with number of features equal to 81. Subsequently, we evaluate the classification performance of the test dataset using the 81 attributes on 100 bootstrap samples. The average value of AUC is equal to 0.84. In contrast to SVM-RFE, the average AUC accuracy on the same bootstrap samples is equal to 0.77 with number of features equal to 91. Fig 7 shows the AUC values of ESVM-RFE and SVM-RFE across the 100 bootstrap samples. Table 5 shows the summary classification performance comparison between ESVM-RFE and SVM-RFE in addition to BIRF.

With respect to the breast3 cancer dataset, both ESVM-RFE and SVM-RFE provides approximately similar AUC accuracies with average value equal to 0.66 calculated using the 100 bootstrap test. The different values of AUCs across the 100 bootstrap samples are shown in Fig 7.

#### Experiments on NCI Dataset

The NCI dataset is processed here by ESVM-RFE which produces a set of features ranked based on their weight in decreasing order. The Leave One Out suggests 125 attributes to be selected from this experiment. The average AUC accuracy of the test dataset evaluated by SVM trained on 100 bootstrap samples is equal to 0.81. Similarly, SVM-RFE provides approximately similar average AUC accuracy of 0.78 with number of features equal to 200 (see Fig 7 and Table 5).

## Conlcusion

We propose a method called Ensemble SVM-RFE to select features from gene expression datasets with the ability to handle the problems accompanying these kinds of datasets such as class-imbalanced data and low number of observations. The present study discusses the potential benefits of involving ensemble methods with SVM-RFE. In this paper, we followed the concept of random forest as an example of ensemble and bagging, and adopted SVM-RFE as a benchmark technique that effectively offered state-of-the-art feature selection methods. This ensemble feature selection algorithm improved the process of biomarker identification and classification performance. Our experiments showed that the construction of ensemble SVM at each iteration of SVM-RFE offered improvement in feature subset selection compared to SVM-RFE. Even though ESVM-RFE will not be able to predict which biomarkers can absolutely describe patient relapse, this strategy which involves selecting down to a small number of features draws a meaningful lists of genomic features ranked according to their contribution to the classifier.

We have evaluated our biomarker identification algorithm on five microarray datasets with a particular focus on childhood leukaemia dataset. This is because we have all the clinical data and outcomes of each patient in addition to the probe set ID and gene annotation. The experiments demonstrated that this method provides improvements in the classification accuracy compared to SVM-RFE and random forest based approach (e.g BIRF) algorithms. To bolster our methodology, we employ SVD for visual analytic with a goal to interrogate this complex and voluminous genomic data. SVD shows that patients with similar biological background are near one another in the low dimensional representation with the selected data.

Our future work is to follow our constructionist data analysis strategy to thoroughly assess a range of clinical paradigms associated with childhood cancer. With more genomic data related to individual patient differences there is more opportunity to identifying individuals with significant actionable differences.

## Supporting Information

S1 Text Childhood Leukaemia Dataset. This dataset contains data for 60 patients with expression values for 22,277 probes. It is generated from the U133A platform and collected from The Children’s Hospital at Westmead.
(CSV)
